# Unpacking family meals: state-of-the-art review critiquing the Western ideals, positioning and promotion of family meals

**DOI:** 10.1093/heapro/daaf004

**Published:** 2025-02-12

**Authors:** Fairley Le Moal, Eloise-kate Litterbach, Katherine Dunn, Kylie Fraser, Celeste C Bouchaud, Georgia Middleton

**Affiliations:** Centre Max Weber UMR 5283, Maison des Sciences de l’Homme Lyon St-Étienne, 14 Avenue Berthelot, 69363 Lyon Cedex 07, France; Australian Centre for Behavioural Research in Diabetes, Suite G01, 15-31 Pelham Street, Carlton, VIC 3053, Australia; School of Exercise and Nutrition Sciences, Queensland University of Technology, GPO Box 2434, Brisbane, QLD 4001, Australia; School of Exercise & Nutrition Sciences, Deakin University, Melbourne Burwood Campus, 221 Burwood Highway, Burwood, VIC 3125, Australia; Faculty of Land and Food Systems, Food Nutrition and Health, The University of British Columbia, 2205 East Mall, Vancouver, BC V6T 1Z4, Canada; Flinders University, College of Nursing and Health Sciences, Caring Futures Institute, Tarntanya, Adelaide, SA 5042, Australia

**Keywords:** family meals, ideals, commensality, children, family, health-promoting environments, public health

## Abstract

Family meals are a familiar concept and are practised in many Western households. While academics have been researching family meals for decades, there is much about the family meal we still do not understand. Meanwhile, the promotion of an ‘ideal’ of family meals across media, health and social discourse ensues. An extensive pool of research has indicated cross-sectional associations between the frequency of family meals and optimal health outcomes. However, evidence surrounding what specifically it is about the family meal that could offer health benefits is limited. Furthermore, family meals carry a level of moral value, evoking pressure for parents to ‘achieve’ a family meal ideal, unattainable for many. Family meals research has traditionally focussed on mothers’ perceptions and roles in family mealtimes. While literature is starting to emerge on the often-overlooked cognitive contribution required to plan and execute family meals, little structural supports exist to streamline these efforts or make them more achievable for contemporary families. The published literature is only starting to include cultural and demographic diversity, making it difficult to understand and promote realistic and feasible family meals across populations. The popular depiction of family meals needs to evolve alongside changes in social norms. Our call to action to address these issues is to draw on existing family meal practices, continue to diversify our investigations, and broaden our definitions and understandings of what a family meal is or could be, and how it should be measured.

## Graphical Abstract

**Graphical Abstract:**
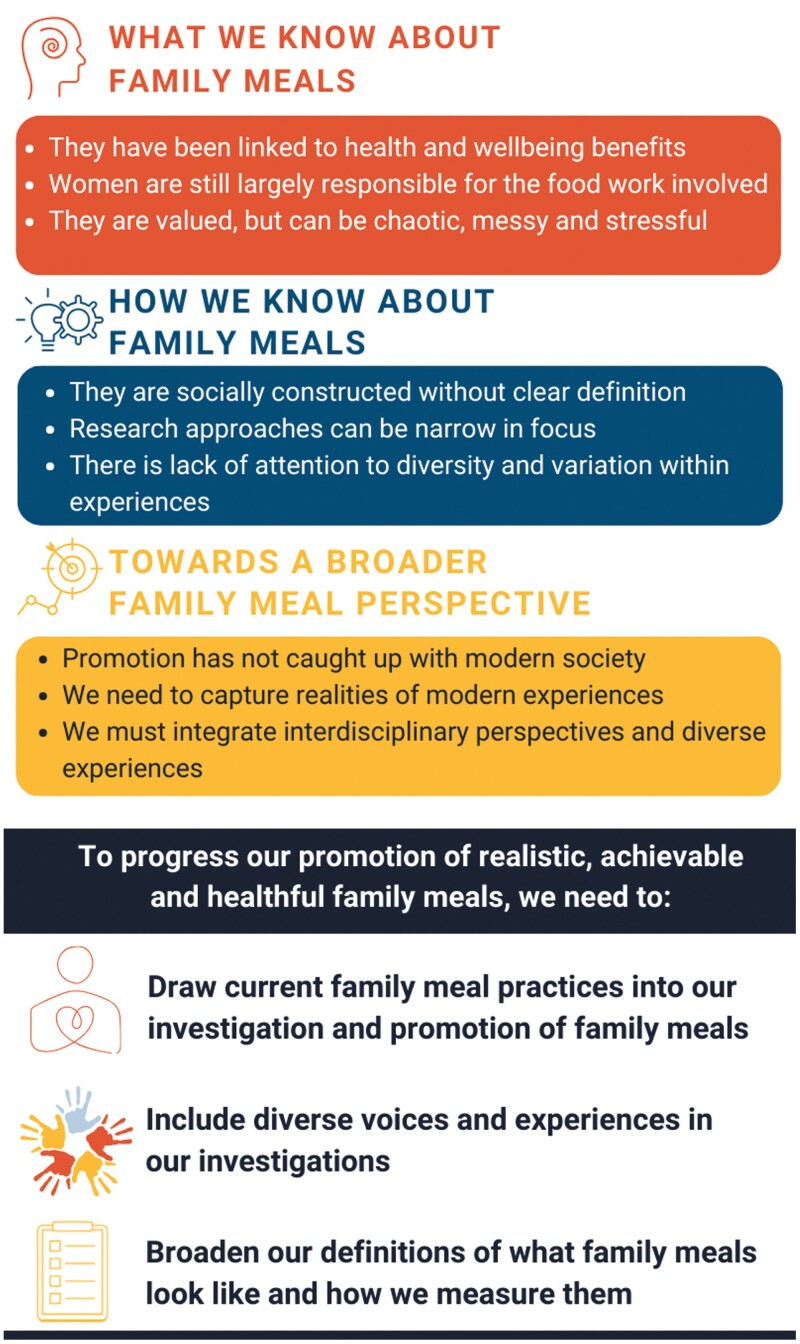

**Contribution to Health Promotion**

- Family meals research has shaped existing health promotion narratives on who, when, where, when and how family meals should take place for optimal child and family health.
- This research is shaped by dominant media and social narratives about family meal ideals, and may reinforce these narratives through current approaches to research in this space.
- This paper presents the case for what we know about family meals, how we came to this knowledge, and where we should go next to promote family meals in healthful, realistic, positive and meaningful ways.

## INTRODUCTION

Sharing a meal together is a uniquely social phenomenon, a core part of human social nature and fundamental in the construction of societies (Elias 1994, Simmel et al. 1997, Fischler 2011). To share a meal together *as a family* has taken on widespread significance, labelled ‘the family meal’ (Marenco 1992). Family meals, along with being a routine practice for many, have been promoted as a positive, family defining activity and increasingly as an avenue for health-promoting behaviours (Kinser 2017, Lindsay et al. 2019, Le Moal et al. 2021). Researchers have thus been seeking to understand their significance and role in family life and implications within society, health and well-being. A challenge surrounding family meals is a commonly held belief that they should include all members of a nuclear family coming together to happily share a nutritious home-cooked meal prepared by the mother. This family meal ideal is perpetuated through social norms, which influence research and health promotion efforts (Andersen and Larsen 2015, Pike and Leahy 2016, Kinser 2017, Le Moal et al. 2021). There is growing concern that continued promotion of such an idealized tradition reinforces unrealistic expectations on today’s resource-pressured families and can lead to perpetuating social inequities.

We acknowledge that our current understanding of family meals is predominantly driven by dominant Western narratives—understood as associated to European, North American and Australasian countries—of the family and of the ways people share food together. In this paper, we discuss (i) what we know about the family meal, (ii) how we have come to that knowledge, and (iii) what is required going forward. In this article, we draw on literature predominantly focussed on families consisting of caregivers (biological or otherwise) and their children between 0 and 18 years.

## WHAT WE KNOW ABOUT FAMILY MEALS

### Family meals may be beneficial for health and wellbeing

According to a recent umbrella review, approximately 25%–50% of families engage in family meals several days a week (Snuggs and Harvey 2023), with higher frequency in families with a stay-at-home mother, young children, and with higher socio-economic positions (Neumark-Sztainer et al. 2010, Dallacker et al. 2018). Academic and mainstream forums highlight that family meals could be an important health-promoting opportunity (Weinstein 2006, Hammons and Fiese 2011), often targeted in childhood and adolescent obesity prevention initiatives (Berge et al. 2015). Beneficial nutrition-related outcomes have been cross-sectionally associated with family meal frequency, including increased fruit and vegetable consumption and weight status (Neumark-Sztainer et al. 2003, Fulkerson et al. 2014, Berge et al. 2015, Dallacker et al. 2018), as well as reduced picky eating and food refusal for younger children (Powell et al. 2017, Cole et al. 2018) and reduced disordered eating behaviours in adolescents (Harrison et al. 2015). Such benefits could stem from the opportunity family meals offer to positively influence children’s eating behaviours through parental modelling, food provision (Campbell et al. 2006, Le Moal et al. 2024) and parental feeding practices (i.e. caregivers goal-directed behaviours that influence what/how much their child eats) (Shloim et al. 2015, Costa and Oliveira 2023).

Considered to enable the construction of family identity (Ochs and Shohet 2006, Jackson 2009, Julier 2013), family meals are an opportunity for caregivers to pass on family values and traditions, strengthen family relationships and create connectedness (Martinasek et al. 2010, Skeer et al. 2018, Utter et al. 2018). They provide a setting for socializing children to diverse skills and behaviours, including table manners, general literacy, language and communication skills and may foster a greater understanding of food and health literacy (Ochs et al. 1989, Snow and Beals 2006, Quick et al. 2011, Malhotra et al. 2013, Quarmby and Dagkas 2013, Le Moal 2024). They also provide a space for discussing issues such as peer relationships and substance use (Skeer and Ballard 2013). Other associations include reduction in adolescent substance use, sexual activity, depression and delinquency (Fisher et al. 2007, Eisenberg et al. 2008).

### Family meals are not always easy or enjoyable

In the context of Western, neoliberal societies, much of the work involved in achieving family meals is undertaken by mothers, even with their increased participation in the paid workforce (Cardon et al. 2019, Craig and Churchill 2021, Meraviglia and Dudka 2021, Seiz 2021, Waddell et al. 2021, Middleton et al. 2023a). Some mothers dislike the traditional allocation of food provision responsibility, others view it is a way to exert power as provider for the family, taking pride over this domain (Lupton 2000, Kavian et al. 2021). This responsibility can lead to paradoxical feelings of satisfaction; providing care and demonstrating love, and conversely, stress and burden (DeVault 1991, Bowen et al. 2019, Fielding-Singh 2021). While many fathers are taking more responsibility for family meals, the division of time, and the mental and emotional investment, is largely still the responsibility of mothers (Fielding-Singh 2017b, Middleton et al. 2023a, Le Moal 2024). Further, the impact of family meals on the health and well-being of caregivers remains understudied (Berge et al. 2012, Tumin and Anderson 2017, Utter et al. 2018). This is a major gap in our understanding, as we may not only be missing the benefits family meals could offer caregivers but missing the negative consequences on caregivers’ time, stress, energy and mental health as a result of pursuing idealized family meals.

Regardless of the dominant maternal labour division of family meals, caregivers report enjoying them (Bugge and Almås 2006, Hooper et al. 2015, Litterbach et al. 2017, Middleton et al. 2023a). This has been influenced by both family meal experiences and widespread representation of happy mealtimes in advertisement, media and public opinion (Oleschuk 2020). Family mealtimes are often portrayed as being fun, relaxing, convivial and loving (Wilk 2010, Phull et al. 2015, Le Moal 2024). However, families face barriers to family meals, including conflicting work, education and recreational schedules, lack of time and/or energy, limited skills or confidence and children’s disruptive behaviour and food refusal at mealtimes (Fulkerson et al. 2011, Berge et al. 2013, Malhotra et al. 2013, Alm and Olsen 2017, Thompson et al. 2021, Middleton et al. 2023a). There are also certain ‘privileges’ that make family meals more, or less, achievable, including physical and mental health, financial security and stability, and space and facilities to safely store, prepare and consume food (Middleton et al. 2023a). Family meals have been described as stressful, chaotic, messy, full of conflict, anxiety and tension (Kling et al. 2009, Meah and Jackson 2013, Rosemond et al. 2019), where issues of power and control can erupt (DeVault 1991, Sjögren 1991, Lupton 1996, 2000, Grieshaber 1997, Kinser 2017, Le Moal 2024). For families with underlying conflicts and violence, family meals are not the site of productive bonding and strengthening of family, but rather fuel for arguments and violence, highlighting family dysfunction (Kling et al. 2009, Rosemond et al. 2019).

## HOW WE KNOW ABOUT ‘FAMILY MEALS’

What we know about family meals has been inherently shaped by the social policies and discourses that shape organization and government priorities, generate ideas and influence funding bodies. To understand what we know about family meals, we must understand how it is we came to gather this knowledge, and what has influenced it.

### Family meal origins and definitions

Family meals while considered a cornerstone of family life, are a socially and historically constructed concept. In Western countries, prior to the twentieth century, family meals did not exist in the conventional sense of how we view them now. It was common for children from upper-class families to eat separately to their parents, and many low-income families did not have the space or resources to feed the whole family at once (Murcott 1997). Academics argue that the family meal was constructed as a bastion against the effects of the industrialization of work on family life, and later as a defence against the liberalization of society (Marenco 1992, Cinotto 2006, Jackson 2009, Bowen et al. 2019). Thus, the ‘traditional’ family meal that is commonly idealized in contemporary society may not have existed without intervention or promotion. Recently, family meals have been positioned as best practice for supporting optimal family health (Government of Canada 2020, LiveLighter 2024, USDA n/d). However, this positioning moralizes family meals as the ‘right thing to do’, offering little structural support to help families achieve them (Oleschuk 2020) and places the responsibility of child health solely on caregivers within their home environments (Ristovski-Slijepcevic et al. 2010, Cappellini et al. 2019, Warin et al. 2019, Warin 2020).

Consequently, there is significant diversity in the way family meals are defined in the literature, with no agreed upon definition of what a ‘family meal’ is (Daragan et al. 2023, Snuggs and Harvey 2023). Defining characteristics may or may not include who is present, the time of day, the location and/or environment, as well as the types of, and how, food is served and consumed (Daragan et al. 2023, Snuggs and Harvey 2023). It is unclear what distinguishes family meals from other eating occasions, and whether it should stand alone. While family meals are defined within the context of individual studies, inconsistency across the literature prevails (DeVault 1991, Martin-Biggers et al. 2014). Reviews have highlighted the difficulty of evaluating the family meal against health outcomes without a standardized definition (Skeer and Ballard 2013, Dwyer et al. 2015, Middleton et al. 2020). The challenge remains that with no clear definition of what makes a ‘family meal’, we are unable to fully comprehend its impact. Yet, enforcing a strict definition of mealtimes could limit our investigations, rather than broaden them.

### Typical approaches to researching family meals

Family meal research has largely been approached using observational and longitudinal study designs. While valuable, these methods cannot provide insight into causal relationships (Musick and Meier 2012, Skafida 2013, Skeer and Ballard 2013, Middleton et al. 2020). Many studies have focussed solely on the nutritional aspects of family mealtimes (Andaya et al. 2011, Appelhans et al. 2014, Avery et al. 2017), others on communication (Ho et al. 2018, Bohn et al. 2024) or on relationships (Armstrong-Carter and Telzer 2020). There is a significant focus on measuring family meal frequency alone (Skeer and Ballard 2013), with more recent investigations into other elements of mealtimes, such as family interactions, atmosphere and environment (Dallacker et al. 2019, Middleton et al. 2020). Qualitative research into family meals has unpacked these components further, looking at the mental, physical and emotional work of planning, procuring, preparing and performing family meals before, during and after the meal (Cappellini and Parsons 2012, Kremer-Sadlik et al. 2015, Lindsay et al. 2019, Dunn et al. 2021, Le Moal et al. 2021, 2024, Fraser et al. 2022, Kremer-Sadlik and Morgenstern 2022, Middleton et al. 2022, Ayre et al. 2023, Litterbach et al. 2023, Le Moal 2024). While intervention trials are emerging in this space, they typically combine targeting family meal components with other sleep, movement and eating behaviour strategies, limiting our ability to determine family meal benefit alone (Middleton et al. 2020).

Furthermore, interdisciplinary studies, for example, where nutritionists and sociologists work together to further our understanding of the significance of family meals in family life and health (Fielding‐Singh and Oleschuk 2023), are essential. Currently, there are two distinctive bodies of work: (i) a public health and nutrition focus on mealtimes themselves and their potential benefits, and (ii) a social sciences focus on the physical, mental and emotional work required to achieve them and how this work affects caregivers (Charles and Kerr 1986, DeVault 1991, Fielding-Singh 2017a, Bowen et al. 2019, Cappellini et al. 2019, Warin et al. 2019, Middleton et al. 2022, Fielding-Singh and Cooper 2023, Le Moal 2024). Eating together as a family involves an interplay of competing values, priorities, preferences and resources, and it is important that these are considered when making investigations and assumptions about mealtime practicalities (Thompson et al. 2016, Middleton et al. 2022, Le Moal 2024). Without interdisciplinary approaches to researching family meals, we are struggling to reconcile the recommendations with the realities of mealtimes.

### Diversity within family meal research

While diversity of research participants is an ongoing challenge across academia, including in family meals research, researchers in this field have been actively including voices other than the dominant white, educated, middle- and upper-class family, predominantly mothers’ perspectives. Particular attention has been paid to families experiencing socio-economic disadvantage unpacking the pressures these families face (Agrawal et al. 2018) and advocating for stronger structural support instead of education-based strategies (Brophy-Herb et al. 2017, Bekelman et al., 2019, Middleton et al. 2023a, 2023b, So et al. 2024a). Research has also focussed on testing our understanding of family meals with racially/ethnically and socioeconomically diverse households (Berge et al., 2016a, 2016b), attempting to understand the role of family meals in the health inequity discrepancies between populations (Campbell et al. 2002, Berge et al. 2018), and developing culturally appropriate interventions (Jarrett et al. 2016). Fathers perspectives are also increasingly being included (Metcalfe et al. 2009, Mallan et al. 2014, Khandpur et al. 2016, Fielding-Singh 2017b, Middleton et al. 2023a, 2023b, Le Moal 2024, So et al. 2024b), providing fundamental understanding of their roles and perspectives, and how we can shift social norms and policies to support their involvement in feeding children.

However, the space is still dominated by Western explorations, and cultural variations remain less explored. For example, research has indicated that parents from certain cultural backgrounds are more likely to pressure children to eat than others (Momin et al. 2014). We know there is a focus on quality, commensality and ‘tradition’ in countries like France, Italy and Switzerland (Fischler 2011), contrasted by a greater emphasis on personal choice and individuality when eating in the USA and UK (Fischler 2011). Family meal timing is also highly varied, with the midday meal most commonly considered the ‘family meal’ in China (prepared by parents or grandparents) and in Norway (Bugge and Almås 2006, Goh 2013), whereas families in Morocco emphasize the snack time between lunch and dinner as a collective eating occasion without the stress and pressure involved in preparing a hot meal (Zirari 2020). These variations are not typically represented in popular media or academia, and should, along with indigenous ways of eating and meaning making through food, be explored and incorporated into our investigations and understandings.

## TOWARDS A BROADER FAMILY MEAL PERSPECTIVE

Existing family meals research has provided thought-provoking ideas for the family meal as a valuable setting for health promotion within the home. A focus going forward requires expanding this understanding of family meals and their potential benefits in depth, breadth and diversity to promote a practice that is feasible and does not contribute to the burden and stressors families already face.

### Moving beyond ideals

It is not surprising that family mealtime behaviours have shifted with changing societal norms and demands. However, our idealization and promotion of family meals in many cases has not. The modern family takes many forms, including blended families, single-headed households, LGBT+ caregivers and intergenerational homes. Many households have caregivers who work non-traditional shifts, have varied work fractions or who work from home. Furthermore, extracurricular schedules of both children and caregivers may overlap with times traditionally set aside for family mealtimes (Brannen et al. 2013, Larson et al. 2020), and the impact of meals provided to children outside of the home (e.g. at school, childcare) on family meal routines is not yet known. Busy, modern families may use convenience foods, eat meals on the go or separately to other family members (Alm and Olsen 2017, Litterbach et al. 2017, Trofholz et al. 2018). Additionally, many caregivers do not have the emotional resources to conduct the food work necessary for family meals to happen every day (Bowen et al. 2019, Fielding-Singh 2021, Le Moal 2024). The idealized family meal is likely not a reality for many families (James et al. 2009, Bowen et al. 2019, Woolhouse et al. 2019, Oleschuk 2020, Fielding-Singh 2021). Research suggests that caregivers strongly value family meals (Litterbach et al. 2017, Middleton et al. 2023a, Le Moal 2024, VicHealth 2024); however, they are also faced with trading off the desire for family meals with the difficulties of executing them (Middleton et al. 2023b). At times, this juggle requires compromise with other healthful activities (Larson et al. 2020). However, against all odds, many caregivers are *trying*. Working with caregivers, drawing on their motivations, values and current practices to understand what they want and need to support *their* family meals is critical.

Family meal research and promotion also need to keep up with advances in society. One clear example is in understanding the evolving role of technology in family life. Many families eat meals while using handheld devices (Robinson et al. 2022) and watching TV (Litterbach et al. 2017), which increases with child age (Boylan et al. 2017). Screen use at mealtimes has been associated with increased distractions, sub-optimal dietary intake, reduced ability to moderate hunger and fullness and stymying social interaction, communication and eating-related development (Avery et al. 2017, Dallacker et al. 2018, Litterbach et al. 2022, 2023, Robinson et al. 2022). However, there is also evidence that points to the complexity of technology use at mealtimes, indicating that screen use can be used to increase children’s vegetable intake, keep children seated and calm and support caregiver’s emotional capacity during the meal (Kaufmann 2011, Schwartz 2012, Ferdous et al. 2016, Wenhold and Harrison 2018, Bekelman et al. 2019, Litterbach et al. 2023, Middleton et al. 2023a). While efforts to promote best practice behaviours at mealtimes should continue, exploring these relationships to inform our recommendations may support safe promotion of family meals without contributing to the guilt and emotional burden many caregivers face when trying to execute family meals. Recommendations informed by research need to strike a balance between *best* practice and *current* practice.

### Expanding our research approach

Family mealtime practices and interactions are highly routinized and often invisible even to those undertaking the work. This makes reporting on these practices and processes difficult. To advance family meals research, we need more investigation into what actually happens leading up to and at family meals, including emotions, conversations, ways of eating and foods consumed, for example, via direct observational methods (Cappellini and Parsons 2012, Bowen et al. 2019, Fielding-Singh 2021, Ayre et al. 2023, Gibson 2023, Le Moal 2024, Le Moal et al. 2024). Further, we need to continue to incorporate cultural, gender and socio-economic diversity within our research to ensure findings and recommendations are applicable to those beyond the perspectives often dominant in health and food research. Expanding our methods to incorporate observations alongside interviews, surveys, and other traditional methods with diverse population groups will help us better understand how the different dimensions of family mealtimes are interconnected.

### Broadening our definitions

Finally, to capture modern family meals our conceptualization should be broadened. Considering alternate versions of family meals that do not involve all family members, take place at a table, occur in the evening, or centre around a home-cooked hot meal would be valuable. Integrating interdisciplinary perspectives of, and approaches to family meals, such as sociology, anthropology, marketing, public health, nutrition, and policy research will help to better understand the family meal in its breadth. Gaining a better understanding of family meals in this way, broadening our understanding of what mealtimes encompass and the potential benefits they may have could support assessment that captures modern family mealtimes in their complexity and is not limiting to time of day, location and physical resources privileged by socio-economic opportunity. Such a focus could contribute to the shift researchers are driving toward a focus on quality of meals over quantity (Dallacker et al. 2019), which may be more achievable for modern families.

## THIS IS OUR CALL TO ACTION

To progress the promotion of realistic, achievable and healthful family meals, we need to:

1. Align with a strengths-based approach by drawing on current family meal practices that people already enact within their homes into our investigation and promotion of family meals,
2. Continue to diversify our investigations beyond the dominant narratives and include diverse voices on advisory committees, as ‘consumer researchers’ and as participants,
3. Broaden our definitions of what family meals look like, what we should measure and how we should measure them to fully capture the complexity and potential benefits of family meals to all family members.

Despite the many critiques and challenges of family meal research and promotion presented in this paper, family meals remain a valued tradition in many Western households. Family meals could remain a unique opportunity for health promotion, if we continue to push boundaries with our explorations, ensuring we are promoting realistic, achievable and healthful practices. Continuing to broaden our understanding of what constitutes a family and what feeding the family means will contribute to reducing the moral imperative around family meals. Furthermore, supporting caregiver’s capacity to have family meals requires advocating for structural change through more flexible workplace policies, family-friendly and nutritionally sound convenience options and community, and school and workplace programs that support preparation or provision of nutritious meals.

## Data Availability

None applicable.
